# Publisher Correction: Development, evaluation of the PNA RT-LAMP assay for rapid molecular detection of SARS-CoV-2

**DOI:** 10.1038/s41598-021-01169-7

**Published:** 2021-11-10

**Authors:** Chinbayar Bat-Ochir, Yeon-Sook Kim, Han Gyeul Kim, Si Seok Lee, Han Woo Lee, Hee Kyung Park

**Affiliations:** 1 Seasun Biomaterials, Daejeon, Korea; 2 Division of Infectious Diseases, Department of Internal Medicine, Chungnam National University School of Medicine, Daejeon, Korea

Correction to: *Scientific Reports* https://doi.org/10.1038/s41598-021-00041-y, published online 14 October 2021

The original version of this Article contained errors in Table 3b, where the heading showing data for “RdRp” and “N gene” was incorrectly given as “Mean Ct (real-time PCR)”. The correct heading is “Mean Tt (RT LAMP)”.

The incorrect and correct values are given below.

Incorrect:
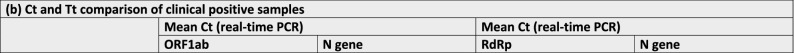


Correct:
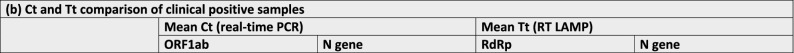


The original Article has been corrected.

